# Communication Impairments in Mice Lacking *Shank1*: Reduced Levels of Ultrasonic Vocalizations and Scent Marking Behavior

**DOI:** 10.1371/journal.pone.0020631

**Published:** 2011-06-09

**Authors:** Markus Wöhr, Florence I. Roullet, Albert Y. Hung, Morgan Sheng, Jacqueline N. Crawley

**Affiliations:** 1 Laboratory of Behavioral Neuroscience, National Institute of Mental Health, Bethesda, Maryland, United States of America; 2 The Picower Institute for Learning and Memory, Massachusetts Institute of Technology, Cambridge, Massachusetts, United States of America; Université de Bordeaux and Centre National de la Recherche Scientifique, France

## Abstract

Autism is a neurodevelopmental disorder with a strong genetic component. Core symptoms are abnormal reciprocal social interactions, qualitative impairments in communication, and repetitive and stereotyped patterns of behavior with restricted interests. Candidate genes for autism include the *SHANK* gene family, as mutations in *SHANK2* and *SHANK3* have been detected in several autistic individuals. *SHANK* genes code for a family of scaffolding proteins located in the postsynaptic density of excitatory synapses. To test the hypothesis that a mutation in *SHANK1* contributes to the symptoms of autism, we evaluated *Shank1* ^−/−^ null mutant mice for behavioral phenotypes with relevance to autism, focusing on social communication. Ultrasonic vocalizations and the deposition of scent marks appear to be two major modes of mouse communication. Our findings revealed evidence for low levels of ultrasonic vocalizations and scent marks in *Shank1* ^−*/*−^ mice as compared to wildtype *Shank1* ^+/+^ littermate controls. *Shank1* ^−*/*−^ pups emitted fewer vocalizations than *Shank1^+/+^* pups when isolated from mother and littermates. In adulthood, genotype affected scent marking behavior in the presence of female urinary pheromones. Adult *Shank1* ^−*/*−^ males deposited fewer scent marks in proximity to female urine than *Shank1^+/+^* males. Call emission in response to female urinary pheromones also differed between genotypes. *Shank1^+/+^* mice changed their calling pattern dependent on previous female interactions, while *Shank1* ^−*/*−^ mice were unaffected, indicating a failure of *Shank1* ^−*/*−^ males to learn from a social experience. The reduced levels of ultrasonic vocalizations and scent marking behavior in *Shank1* ^−*/*−^ mice are consistent with a phenotype relevant to social communication deficits in autism.

## Introduction

Autism is a neurodevelopmental disorder characterized by abnormal reciprocal social interactions, deficits in social communication, motor stereotypies, repetitive behaviors, and narrow restricted interests [1]. While the causes of autism remain unknown, the high concordance between monozygotic twins supports a strong genetic component [2], [3]. Genome-wide and pathway-based association studies led to the identification of several susceptibility genes for autism, including the *SHANK* gene family [4], [5]. Mutations in *SHANK3* and deletions in the chromosome 22q13.2 region containing *SHANK3* have been described in autistic patients [6]–[9]. Mutations in *SHANK2* were recently reported in several individuals with autism [10], [11].

*SHANK* genes code for a family of multidomain scaffolding proteins located in the postsynaptic density (PSD) of glutamatergic synapses [12]–[14]. SHANK proteins anchor NMDA, AMPA, and metabotropic glutamate receptors in the postsynaptic membrane, connecting them with signaling proteins and the actin cytoskeleton, assembling G-protein-mediated signaling and regulating calcium homeostasis in dendritic spines [15]–[19]. By virtue of their central position within the PSD, Shank proteins were termed “master scaffolding proteins” [13]–[14]. In addition, they promote morphological and functional maturation of dendritic spines and synapse formation. As shown in overexpression experiments, increased levels of Shank led to an enlargement of dendritic spines through the enhanced recruitment of Homer to the postsynaptic site [20]. Three Shank isoforms are currently known in mice, Shank1, Shank2, and Shank3. All of them are characterized by multiple ankyrin repeats, followed by SH3, PDZ, a long proline-rich region and a C-terminal sterile alpha motif [12]–[14]. Due to their structural similarity, most Shank interaction partners such as Homer or GKAP are equally recognized by all three isoforms [17], [21], indicating similar physiological roles.

Hung et al. [22] disrupted the *Shank1* gene in mice, to investigate its function in vivo. *Shank1* ^−*/*−^ mutant mice showed altered protein composition of the PSD with reduced levels for Shank, Homer, and GKAP. Dendritic spines and synapses were smaller, which correlated with weakening of excitatory synaptic transmission. Behaviorally, *Shank1* ^−*/*−^ mice displayed reduced locomotion, impaired rotarod performance, higher anxiety-like behavior, but normal levels of social interactions [22], [23]. In addition, Hung et al. [22] observed impaired contextual fear conditioning, normal cued fear conditioning, and enhanced acquisition but impaired retention of spatial learning in *Shank1* ^−*/*−^ mice. As suggested by Hung et al. [22], these behavioral phenotypes might be reminiscent of the heterogeneous cognitive phenotypes seen in people with autism.

To test the hypothesis that a mutation in *SHANK1* contributes to the symptoms of autism, we evaluated *Shank1* ^−/−^ null mutant, *Shank1^+/^* ^−^ heterozygote, and *Shank1^+/+^* wildtype littermate control mice for behavioral phenotypes with relevance to autism. Here, we focus on communication deficits in mice that may incorporate conceptual analogies to the qualitative impairments in communication such as delayed language and poor communication skills, which are fundamental to the diagnosis of autism [1], [24]–[27]. Mice communicate predominantly via acoustic [28]–[31] and olfactory signals [32]–[34]. Zippelius and Schleidt [35] discovered that mouse pups emit ultrasonic vocalizations (USV) when isolated from their mother and littermates. Supporting a communicative function, isolation-induced USV elicit maternal search and retrieval behavior, as shown in playback experiments [36]–[41]. Besides early environmental factors, USV production in pups is strongly dependent on genetic background, as shown by genetic analyses using reciprocal hybrids [42]–[47] and embryo-transfer [41]. Reduced levels of pup USV or unusual calling patterns were detected in several genetic mouse models of autism [48]–[62].

In adult mice, high USV levels are detected in males when courting and copulating with females [63]. Female urine alone, i.e. in the absence of a female mouse, is sufficient for eliciting USV in males [64]–[77]. USV emission in males exposed to female urine is highly sensitive to important social factors such as previous social experience [64], [65], [67], [69]–[73], but not on the females' estrus cycle [29], [77]. Female-induced USV appear to serve an important communicative function, namely to attract females, as shown in devocalization studies and playback experiments [78], [79]. Several mouse models of autism were reported to display reduced levels of adult male USV production in response to females or female urine [76], [80]–[82].

In addition to USV, adult male mice display scent marking behavior, depositing urinary pheromone traces in close proximity to the location of female urine [71], [76], [83]–[87]. In support of a communicative function, scent marks by adult male mice function as a negative advertisement to exclude other adult males from the territory and hence prevent potential competition for females [88], [89] and as a positive advertisement directed towards females for attraction of mates [90–95)]. Olfactory communication was only rarely evaluated in mouse models of autism. The BTBR T+tf/J inbred strain mouse model of autism displayed reduced levels of scent marking behavior and an almost complete lack of USV [76], supporting the simultaneous evaluation of these two phenotypes. The present studies tested *Shank1* ^−*/*−^ mutant mice for developmental milestones and pup isolation-induced USV, and in our assays for adult male scent marking and USV to female urine in an open field. Results are consistent with an interpretation of communication deficits relevant to autism.

## Materials and Methods

### Animals and housing

*Shank1* ^−*/*−^ null mutant mice with a targeted replacement of exons 14 and 15 encoding almost the entire PDZ domain were compared to *Shank1^+/^* ^−^ and *Shank1^+/+^* littermate control mice. Mice were obtained from mutant lines originally generated by Hung et al. [22] on two independent background strains: C57BL/6J and 129SvJae. As high mortality rates were obtained in the C57BL/6J background strain and very low locomotion in the 129SvJae background strain [23], the two lines were crossed for at least three generations to produce a mixed C57BL/6J/129SvJae background for the *Shank1* mutation, consistent with the other studies on *Shank1* mutants [22], [23]. Using a heterozygous breeding protocol, *Shank1^+/^* ^−^ males and females were bred in a conventional vivarium at the National Institute of Mental Health in Bethesda, MD, USA. Approximately 2 weeks after pairing for breeding, females were individually housed and inspected daily for pregnancy and delivery. The day of birth was considered as postnatal day (pnd) 0. After weaning on pnd 21, mice were socially housed in groups of 2–4 with same-sex partners. All mice were housed in polycarbonate Makrolon IVC cages (369×156×132 mm, 435 cm^2^; 1145T; Tecniplast, Milan, Italy). Bedding, paper strips, a nestlet square and a cardboard tube were provided in each cage. Standard rodent chow and water were available ad libitum. The colony room was maintained on a 12∶12 light/dark cycle with lights on at 06∶00 h, at approximately 20°C and 55% humidity. Pups were identified by paw tattoo, using non-toxic animal tattoo ink (Ketchum permanent Tattoo Inks green paste, Ketchum Manufacturing Inc., Brockville, ON, Canada). The ink was inserted subcutaneously through a 30 gauge hypodermic needle tip into the center of the paw. All procedures were conducted in strict compliance with the National Institutes of Health Guidelines for the Care and Use of Laboratory Animals and approved by the National Institute of Mental Health Animal Care and Use Committee.

### General overview

Two independent cohorts of mice were tested to avoid potential confounds from using previously handled animals. Mice of Cohort 1 (4 litters; 9.50±1.19 pups/litter; mean±SEM) were tested for developmental milestones from pnd 2–12. Cohort 1 consisted of n = 9 *Shank1^+/+^* littermate control mice (females: n = 6; males: n = 3), n = 11 *Shank1^+/^* ^−^ (females: n = 6; males: n = 5), and n = 16 *Shank1* ^−*/*−^ mutant mice (females: n = 11; males: n = 5). Mice of cohort 2 were tested for USV in isolation on pnd 8. After measuring pup USV, body weight and body temperature were determined. At the age of 13.00±0.50 weeks, adult male mice were tested for open field activity and basal scent marking behavior when exposed to a clean open field for 60 min. Immediately thereafter, a drop of female urine was added to the open field. Open field activity, female urine elicited scent marking behavior and USV were scored during the 5 min exposure to the female urine. Approximately 7 days later, adult male mice were exposed to females for 5 min. This was the first inter-sexual contact after weaning. Approximately 7 days after female experience, adult male mice were exposed again first to the clean open field for 60 min and then for 5 min to the female urine. A subgroup of cohort 2 was used to measure body weight at approximately 5 months of age. Mice of cohort 2 were obtained from mothers that gave birth twice, named first litter mice from primiparous females (13 litters; 9.23±0.70 pups/litter) and second litter mice from multiparous females (7 litters; 9.57±0.65 pups/litter). Cohort 2 consisted of n = 42 *Shank1^+/+^* littermate control mice (first litter females n = 13; second litter females n = 10; first litter males n = 13; second litter males n = 6), n = 44 *Shank1^+/^* ^−^ (first litter females n = 14; second litter females n = 7; first litter males n = 15; second litter males n = 8), and n = 54 *Shank1* ^−*/*−^ mutant mice (first litter females n = 15; second litter females n = 14; first litter males n = 14; second litter males n = 11). A heterozygous breeding protocol was used throughout. About half of the *Shank1^+/^* ^−^ mutant mice were randomly excluded from the study to obtain similar numbers of mice per genotype.

### Developmental milestones and somatosensory reflexes

Pups of Cohort 1 were tested according to a modified Fox battery for developmental milestones and somatosensory reflexes [96], [97]. The tests were conducted between 09:00–14:00 h during the light phase of the 12∶12 h light/dark cycle. Each subject was tested at approximately the same time of day. Every other day from pnd 2–12, body weight, length, and temperature were measured. Body weight was measured using a palmscale (PS6-250; My Weigh Europe, Hückelhoven, Germany). For body temperature determination a DiGiSense Thermistor Thermometer (Thermo Fisher Scientific Inc., Waltham, MA, USA) was used. The following physical landmarks were also recorded: Pinnae detachment, eye opening, incisor eruption, and fur development. Somatosensory reflexes and responses were scored in the following order:

1. Surface righting: The pup is gently held on its back and released. Latency to flip over onto the abdomen with four paws touching the surface is measured with a stopwatch.
2. Negative geotaxis: The pup is gently placed head down on a square of grid (8×11 cm) at an angle of 45°. Latency to turn 180° to either side is measured with a stopwatch.
3. Cliff avoidance: The pup's snout and forepaws are gently pushed over the edge of a table. Latency to withdraw from the edge of a flat surface is measured with a stopwatch.
4. Grasping reflex: The pup's paw is stroked with a toothpick. Grasping the shaft of the toothpick is recorded as present or absent.
5. Level screen holding: The pup is dragged across a square grid (8×11 cm) by the tail. Grasping is recorded as present or absent.
6. Vertical screen holding: The pup is placed on a square grid (8×11 cm) at 90° angle. Length of time the pup is able to stay on the grid is measured with a stopwatch.
7. Bar holding: The pup grasps a small elevated wire bar by its forelimbs while the hindlimbs are not in contact with the surface. Length of time the pup is able to hold onto a bar is measured with a stopwatch.
8. Auditory startle: The pup is exposed to an acoustic stimulus (hand clapping). Startle response is recorded as present or absent.

Latencies were measured in seconds for surface righting (maximum: 60 s), negative geotaxis (maximum: 60 s), cliff avoidance (maximum: 60 s), vertical screen holding (maximum: 10 s) and bar holding (maximum: 10 s). Other somatic and behavioral variables were rated semi-quantitatively, 0 =  no response/not present, 1 =  slight response/slightly present, 2 =  strong response/strongly present, 3 =  incomplete response/incompletely present, and 4 =  complete adult-like response/presence. Experimenters were trained until the inter-observer reliability was greater than 95%.

### Ultrasonic vocalizations in isolated pups

Pups of Cohort 2 were isolated from their mother and littermates on pnd 8 for 5 min under room temperature (23–24°C). Pups were removed individually from the nest at random and gently placed into an isolation container (10×8×7 cm; open surface) made of glass, containing clean bedding material. The isolation container was surrounded by a sound attenuating box (18×18×18 cm) made of Styrofoam (thickness of walls: 4 cm). USV emission was monitored by an UltraSoundGate Condenser Microphone CM 16 (Avisoft Bioacoustics, Berlin, Germany) placed in the roof of the sound attenuating box, 10 cm above the floor. The microphone was connected via an UltraSoundGate 116 USB audio device (Avisoft Bioacoustics) to a personal computer, where acoustic data were recorded with a sampling rate of 250,000 Hz in 16 bit format by Avisoft RECORDER (version 2.97; Avisoft Bioacoustics). The microphone that was used for recording was sensitive to frequencies of 15–180 kHz with a flat frequency response (±6 dB) between 25–140 kHz. After the 5 min isolation period, body weight and body temperature were determined as described above. Isolation occurred between 8.00–16.00 h during the light phase of the 12∶12 h light/dark cycle. Prior to each test, behavioral equipment was cleaned using a 70% ethanol solution, followed by water, and dried with paper towels.

**Figure 1:**
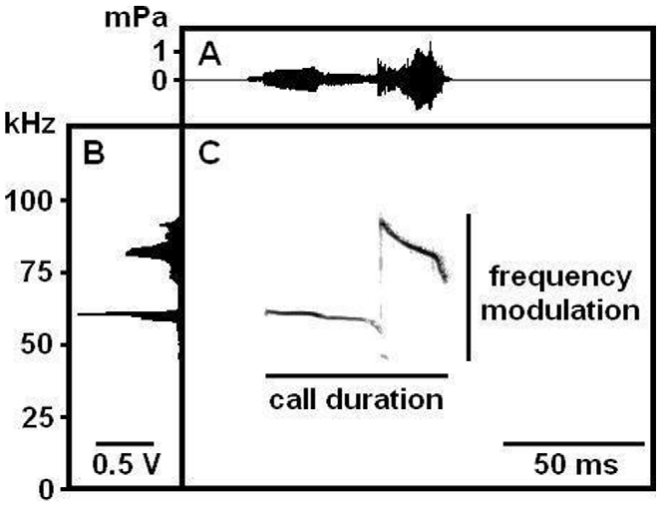
Analysis of ultrasonic vocalizations. Energy within the spectrum is shown by time (A) and frequency (B) and time x frequency (C, reflected as “darkness”). Peak amplitude, i.e. loudness, was defined as the point with the highest energy within the spectrum (“darkest” points over time in C). Peak frequency was defined as the frequency at the location of the peak amplitude within the spectrum (“darkest” points over time in C). Peak frequency and peak amplitude were derived from the average spectrum of the entire call, meaning that values obtained per time point were averaged over time. The extent of frequency modulation was defined as the difference between the lowest and the highest peak frequency within each call, i.e. derived from the non-averaged call (C). Temporal parameters were latency to start calling, total calling time, call duration (C).

For acoustical analysis, recordings were transferred to Avisoft SASLab Pro (version 4.50; Avisoft Bioacoustics) and a fast Fourier transform was conducted (512 FFT length, 100% frame, Hamming window and 75% time window overlap). Correspondingly, the spectrograms were produced at 488 Hz of frequency resolution and 0.512 ms of time resolution. Call detection was provided by an automatic threshold-based algorithm (amplitude threshold:−40 dB) and a hold-time mechanism (hold time: 10 ms). Since no USV were detected below 30 kHz, a high-pass filter of 30 kHz was used to reduce background noise outside the relevant frequency band to 0 dB. The accuracy of call detection by the software was verified manually by an experienced user. When necessary, missed calls were marked by hand to be included in the automatic parameter analysis. Total number of USV was calculated for the entire session and in 60 s time bins, to visualize the time course of the USV response. Additional parameters, based on previous studies of isolation-induced calling [41], [98], [99], included peak frequency and peak amplitude, i.e. loudness, which were derived from the average spectrum of the entire call, were determined automatically (Fig. 1). Peak amplitude was defined as the point with the highest energy within the spectrum. Peak frequency was defined as the frequency at the location of the peak amplitude within the spectrum. In addition, the extent of frequency modulation, i.e. the difference between the lowest and the highest peak frequency within each call, was measured automatically. Temporal parameters included latency to start calling, total calling time, and call duration.

### Open field activity, scent marking behavior, and ultrasonic vocalizations in the absence and presence of female urine in adult males

Scent marking behavior and USV in adult male mice of Cohort 2 were recorded during a 5 min session of exposure to a drop of fresh female urine in the center of an open field (40×49×30 cm) as previously described [71], [76]. Urinary scent marks, USV, and open field activity were scored within the same test session. All mice were tested twice, at time points separated by approximately 14 days. After the first but before the second test session, each male subject mouse was exposed to an unfamiliar C57BL/6J female. Testing occurred between 10.00–17.00 h during the light phase of the 12∶12 h light/dark cycle.

#### Urine collection

Adult male mice were exposed to fresh urine obtained from randomly-selected C57BL/6J females. Urine was collected from adult females in estrus by using a method adopted from Nyby et al. [100] as previously described [71], [76]. Briefly, female donor mice were socially housed in groups 2–4 and were approximately 2–3 months of age at the time of urine collection. The donor female was gently taken out of its home cage by the tail and held by the base of the tail on the home cage lid. Gentle pressure was applied to lift the back and expose the genital area in order to determine the phase of the estrus cycle. The female was scored as in estrus when the vaginal area appeared opened and red [71], [76]. The act of handling the female in this manner was usually sufficient to cause it to urinate. Urine was collected in a 1.0 ml Eppendorf tube. 15 µl of fresh female urine was immediately pipetted into the center of the open field.

#### Female experience

In order to provide a standardized prior history of social experience, each adult male mouse was individually placed with a randomly-selected adult C57BL/6J female for 5 min in a clean polycarbonate Makrolon cage (369×156×132 mm, 435 cm^2^; 1145T; Tecniplast) containing clean bedding, approximately 7 days after the first and approximately 7 days before the second test session. Multiple females were used in order to minimize the number of male exposures and hence aggressive behavior against males.

#### Test procedure

Adult male mice were individually habituated for 60 min to the clean open field lined with a sheet of specialized paper (Strathmore Drawing Paper Premium, recycled, microperforated, 400 series; Strathmore Artist Papers, Neenah, WI, USA) that effectively absorbed drops of mouse urine, and containing some of their own home cage bedding in one corner of the arena to reduce the stress of the novel open field. At the end of the habituation period, the subject mouse was placed back in a clean polycarbonate Makrolon cage (369×156×132 mm, 435 cm^2^; 1145T; Tecniplast) with fresh bedding. The home cage bedding and any feces deposited during the habituation session were removed from the open field. Scent marks deposited on the paper during the habituation session were visualized under ultraviolet (UV) light using a UV lamp (Sleeklook Super 18” Black Light-eParty unlimited; Can You Imagine, Chatsworth, CA, USA). Visualized scent marks were outlined using a pencil. 15 µl of female urine was then pipetted onto the center of the Strathmore paper, and the mice were placed back into the open field for 5 min. The second set of scent marks deposited on the paper during the 5 min exposure to the female urine was visualized under the UV lamp and outlined with a blue colored pen. Prior to each session with a new subject mouse, the open field was cleaned with a 70% ethanol solution, followed by water, and dried with paper towels. The entire habituation and testing procedure was performed twice. Test 1 was conducted before males had female experience. Approximately 7 days later, males were exposed to females for five minutes, as described above. Test 2 was conducted approximately 7 days after the 5 minute interaction with a female. Open field activity, scent marking behavior and USV were scored during both test 1 and test 2. Habituation and testing was performed under red light.

#### Open field activity

Locomotor activity was automatically recorded by a Versamax animal activity monitor (AccuScan Instruments, Inc., Columbus, OH, USA) during the 60 min habituation session to the clean open field without female urine and during the 5 min test session with exposure to female urine. Time spent within an area of 10 cm^2^ surrounding the female mouse urine spot was also recorded.

#### Scent marking behavior

Urinary scent marks were scored as previously described [32], [71], [76], [101]. Briefly, at the end of each female urine exposure, the marked sheets of Strathmore paper were treated with Ninhydrin spray (LC-NIN-16; TritechForensics Inc., Southport, NC, USA) then left to dry for about 12 h, which allowed the visualization of the urine traces as purple spots. For counting of scent marks, a transparent plastic grid (40 cm^2^) divided into squares, 1 cm^2^ per square, was placed on the top of the sheet of Strathmore paper. The total number of scent marks and the number of scent marks within an area of 10 cm^2^ around the female urine spot were counted. Scent marks deposited during the 60 min habituation session and the 5 min test session with exposure to female urine were differentiated based on their visualization with pencil or blue colored pen, respectively.

#### Ultrasonic vocalizations

USV emission was recorded with a sampling rate of 300,000 Hz in 16 bit format by Avisoft RECORDER (version 2.97; Avisoft Bioacoustics) as previously described [71], [76]. For acoustical analysis, a fast Fourier transform was conducted as described above. An experienced observer counted the total number of USV as well as their numbers in 10 s time bins to visualize the time course of the USV response.

### Statistical analysis

Developmental milestones were compared between *Shank1* ^−*/*−^ null mutant, *Shank1^+/^* ^−^heterozygote and *Shank1^+/+^* littermate control mice with ANOVAs for Repeated Measurements. Between-subject factors were genotype and sex. The within-subject factor was age. For analysis of pup USV emitted in isolation, ANOVAs with between-subjects factors of genotype, sex, and parity, i.e. first versus second litter, were calculated. In order to test whether differences in pup USV emitted in isolation emerged over time during testing, ANOVAs for Repeated Measurements with the same between-subject factors and the within-subject factor test duration were calculated. Three outliers were removed from the data set as their values for all parameters determined deviated by >2 standard deviations from the group mean. For analysis of male open field activity, scent marking behavior, and USV in response to female urine, ANOVAs for Repeated Measurements with the between-subject factor genotype and the within-subject factor female experience were calculated. In order to test whether differences in USV emitted by males in response to female urine emerged across testing, ANOVAs for Repeated Measurements with the between-subject factor genotype and the within-subject factors female experience and test duration were calculated. ANOVAs were followed by Bonferroni or Tukey's post hoc analysis when appropriate. A p-value of <0.050 was considered statistically significant.

## Results

### Developmental milestones and somatosensory reflexes

All developmental milestones and somatosensory reflexes varied with age (all p-values <0.050; Table 1 & 2), as expected, with the exception of auditory startle, where a trend was observed (all p-values >0.050 and <0.100).

**Table 1 pone-0020631-t001:** Developmental milestones in *Shank1* mice.

Developmental milestones		pnd 2	pnd4	pnd6	pnd8	pnd10	pnd12
Body weight [g] * ^,$^	**+/+**	1.86±0.07	2.86±0.12	4.04±0.12	5.24±0.16	6.21±0.18	7.28±0.24
	**+/−**	1.72±0.08	2.78±0.11	3.86±0.14	4.92±0.17	5.93±0.20	6.74±0.28
	**−/−**	1.82±0.06	2.78±0.10	3.83±0.10	4.87±0.13	5.86±0.15	6.76±0.19
Body length [cm] *	**+/+**	3.31±0.09	3.82±0.05	4.34±0.06	4.86±0.07	5.06±0.06	5.39±0.07
	**+/−**	3.28±0.05	3.74±0.06	4.23±0.05	4.70±0.06	4.98±0.06	5.17±0.07
	**−/−**	3.34±0.04	3.78±0.05	4.28±0.06	4.73±0.06	4.98±0.06	5.28±0.07
Body temperature [°C] *	**+/+**	39.07±0.35	39.53±0.15	39.98±0.11	40.21±0.11	40.30±0.10	40.67±0.15
	**+/−**	39.42±0.14	39.75±0.12	39.93±0.14	40.08±0.08	40.24±0.07	40.56±0.09
	**−/−**	39.91±0.23	39.29±0.14	39.81±0.11	39.91±0.09	40.24±0.09	40.46±0.11
Pinnae detachment [n] *^,^ ^+^	**+/+**	0.00±0.00	1.89±0.11	1.89±0.11	2.00±0.00	2.00±0.00	2.00±0.00
	**+/−**	0.00±0.00	1.90±0.06	1.90±0.06	2.00±0.00	2.00±0.00	2.00±0.00
	**−/−**	0.00±0.00	1.69±0.12	1.69±0.12	2.00±0.00	2.00±0.00	2.00±0.00
Eye opening [n] *	**+/+**	0.00±0.00	0.00±0.00	0.94±0.06	0.94±0.06	1.78±0.15	2.22±0.22
	**+/−**	0.00±0.00	0.00±0.00	0.90±0.06	0.95±0.03	1.76±0.10	2.48±0.18
	**−/−**	0.00±0.00	0.00±0.00	0.84±0.06	0.84±0.06	1.69±0.15	2.41±0.19
Incisor eruption [n] *^,^ ^+^	**+/+**	0.00±0.00	0.00±0.00	0.50±0.17	1.22±0.22	2.00±0.17	2.94±0.18
	**+/−**	0.00±0.00	0.00±0.00	0.48±0.10	1.19±0.11	1.90±0.17	2.62±0.13
	**−/−**	0.00±0.00	0.00±0.00	0.47±0.11	0.97±0.15	2.41±0.12	2.63±0.13
Fur development [n] *	**+/+**	0.00±0.00	0.03±0.03	0.48±0.03	1.06±0.06	2.53±0.03	3.39±0.07
	**+/−**	0.00±0.00	0.03±0.02	0.48±0.02	1.05±0.03	2.45±0.03	3.31±0.06
	**−/−**	0.00±0.00	0.06±0.03	0.41±0.04	1.08±0.08	2.50±0.06	3.31±0.06

[Table pone-0020631-t001]Data are expressed as means±SEM. PND =  postnatal day. [n] =  semi-quantitative rating (0 =  no response/not present, 1 =  slight response/slightly present, 2 =  strong response/strongly present, 3 =  incomplete response/incompletely present, and 4 =  complete adult-like response/presence). Effect of age: * p<0.050. Effect of genotype: # p<0.050. Effect of sex: NS. Interaction genotype x age: + p<0.050. Interaction sex x age: § p<0.050. Interaction genotype, sex and age: $ p<0.050. +/+ N = 9, +/− N = 11, −/− N = 16.

**Table 2 pone-0020631-t002:** Somatosensory reflexes in *Shank1* mice during early development.

Somatosensory reflexes		pnd 2	pnd4	pnd6	pnd8	pnd10	pnd12
Surface righting [s] *^,#^	**+/+**	8.33±1.75	13.43±2.43	6.29±2.49	3.41±0.72	1.34±0.41	1.12±0.17
	**+/−**	9.52±2.64	11.10±3.62	9.07±2.42	2.93±0.41	1.11±0.12	0.93±0.07
	**−/−**	19.73±4.91	23.56±6.45	18.73±5.77	3.26±0.35	1.43±0.19	1.01±0.15
Negative geotaxis [s] *	**+/+**	7.09±3.00	7.06±1.70	6.86±2.05	8.14±4.02	14.35±3.36	8.60±1.66
	**+/−**	22.09±5.50	21.48±5.22	11.05±3.07	10.21±3.28	6.99±1.53	6.46±0.94
	−**/−**	25.85±7.00	27.25±6.66	9.16±3.60	6.30±1.23	5.27±1.14	4.47±0.78
Bar holding [s] *^,^ ^§^	**+/+**	0.00±0.00	0.00±0.00	0.00±0.00	0.33±0.17	2.44±1.06	6.89±1.06
	**+/−**	0.00±0.00	0.00±0.00	0.00±0.00	0.29±0.16	3.14±0.81	4.69±0.87
	−**/−**	0.00±0.00	0.00±0.00	0.00±0.00	0.50±0.44	3.25±1.09	4.07±0.83
Level screen holding [n] *	**+/+**	0.00±0.00	0.00±0.00	0.00±0.00	1.00±0.00	2.44±0.18	3.00±0.00
	**+/−**	0.00±0.00	0.00±0.00	0.10±0.07	0.90±0.07	2.10±0.14	2.76±0.14
	**−/−**	0.00±0.00	0.00±0.00	0.63±0.04	1.00±0.00	2.06±0.17	2.69±0.12
Vertical screen holding [s] *	**+/+**	0.00±0.00	0.00±0.00	0.00±0.00	6.44±1.08	7.67±0.99	10.00±0.00
	**+/−**	0.00±0.00	0.00±0.00	0.05±0.05	5.17±0.80	6.77±0.76	9.81±0.19
	**−/−**	0.00±0.00	0.00±0.00	0.25±0.12	3.70±0.70	5.71±0.96	8.21±0.75
Grasping reflex [n] *	**+/+**	1.44±0.24	1.78±0.15	2.44±0.18	3.44±0.17	3.89±0.11	3.89±0.11
	**+/−**	1.33±0.17	1.81±0.11	2.67±0.13	3.38±0.13	3.76±0.14	3.86±0.08
	**−/−**	1.44±0.18	1.69±0.12	2.56±0.13	3.06±0.17	3.69±0.12	3.75±0.11
Cliff avoidance [s] *	**+/+**	35.22±9.83	27.22±8.30	5.00±1.54	2.11±0.39	2.44±0.38	2.78±0.92
	**+/−**	34.31±6.14	25.76±5.49	3.19±0.72	3.29±0.44	2.76±0.24	2.24±0.36
	**−/−**	40.04±6.80	41.06±6.44	10.91±4.84	2.81±0.44	2.31±0.31	6.06±3.60
**Auditory startle [n]**	**+/+**	0.56±0.24	0.44±0.18	0.56±0.24	0.67±0.17	0.11±0.11	0.22±0.15
	**+/−**	0.38±0.15	0.38±0.13	0.43±0.13	0.48±0.11	0.33±0.16	0.29±0.10
	**−/−**	0.75±0.31	0.31±0.15	0.38±0.13	0.31±0.12	0.19±0.10	0.13±0.09

[Table pone-0020631-t002]Data are expressed as means±SEM. PND =  postnatal day. [n] =  semi-quantitative rating (0 =  no response/not present, 1 =  slight response/slightly present, 2 =  strong response/strongly present, 3 =  incomplete response/incompletely present, and 4 =  complete adult-like response/presence). Effect of age: * p<0.050. Effect of genotype: # p<0.050. Effect of sex: NS. Interaction genotype x age: + p<0.050. Interaction sex x age: § p<0.050. Interaction genotype, sex and age: $ p<0.050. +/+ N = 9, +/− N = 11, −/− N = 16.

No genotype differences were detected on body weight, length, and temperature (all p-values >0.050). Emergence of physical developmental milestones, however, was affected by genotype. An interaction between genotype and age was found for pinnae detachment (F_10,200_ = 1.954, p = 0.040) and incisor eruption (F_10,200_ = 2.430, p = 0.040; all other p-values >0.050) as both were delayed in *Shank1* ^−*/*−^ null mutant pups. Among somatosensory reflexes, a genotype effect was found for surface righting (F_2,40_ = 5.647, p = 0.007). When gently held on its back and released, it took *Shank1* ^−*/*−^ null mutant pups longer to flip over onto the abdomen with four paws touching the surface than *Shank1^+/^* ^−^ (p = 0.006) and *Shank1^+/+^* littermate control pups (p = 0.030), while the latter did not differ (p = 0.998).

Males and female pups did not differ on developmental milestones (all p-values >0.050), with the exception of bar holding, where an interaction between sex and age was found as males displayed an accelerated development of their bar holding capabilities than females (F_5,200_ = 2.389, p = 0.039). Finally, there was an interaction between genotype, sex, and age (F_10,200_ = 2.345, p = 0.012) as body weight gain was delayed in *Shank1* ^−*/*−^ null mutant female, but not male mice.

### Ultrasonic vocalizations in isolated pups

**Figure 2:**
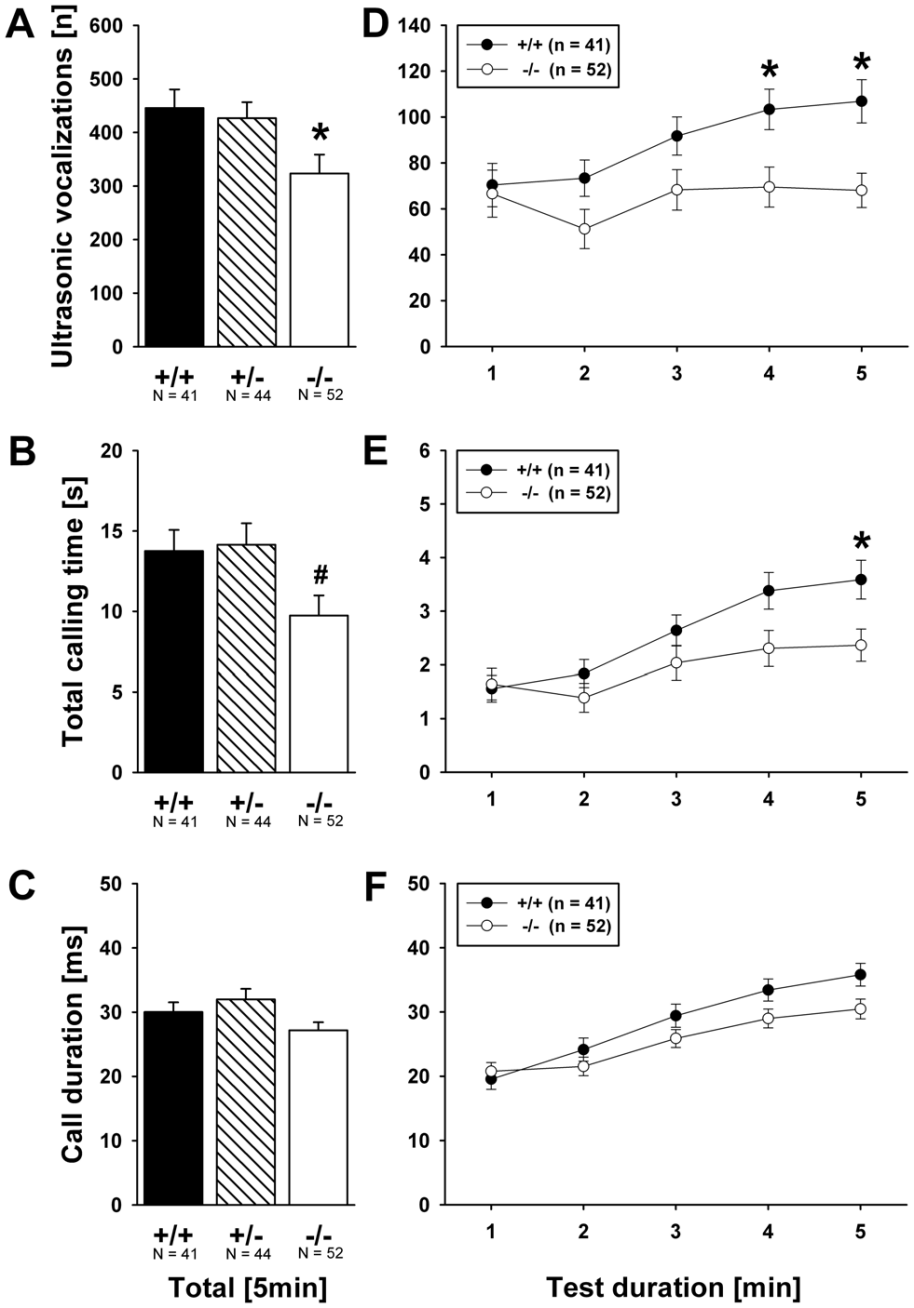
Ultrasonic vocalizations in isolated *Shank1* pups. (A) Total number of ultrasonic vocalizations, (B) total calling time and (C) duration of calls emitted during the 5 min isolation from mother and littermates. (D) Time course for the number of ultrasonic vocalizations, (E) total calling time and (F) duration of calls emitted for each 1 min time bin across the 5 min isolation session. Black bar: *Shank1^+/+^* wildtype littermate control mice; striped bar: *Shank1^+/^* ^−^ heterozygote mice; white bar: *Shank1* ^−*/*−^ null mutant mice. For the sake of clarity, *Shank1^+/^* ^−^ heterozygote mice were not included in the time course graphs, while still included in the statistical analysis. Data are presented as means ± standard errors of the mean. * p<0.050 vs. *Shank1^+/+^*; # p<0.050 vs. *Shank1^+/^* ^−^.

**Figure 3:**
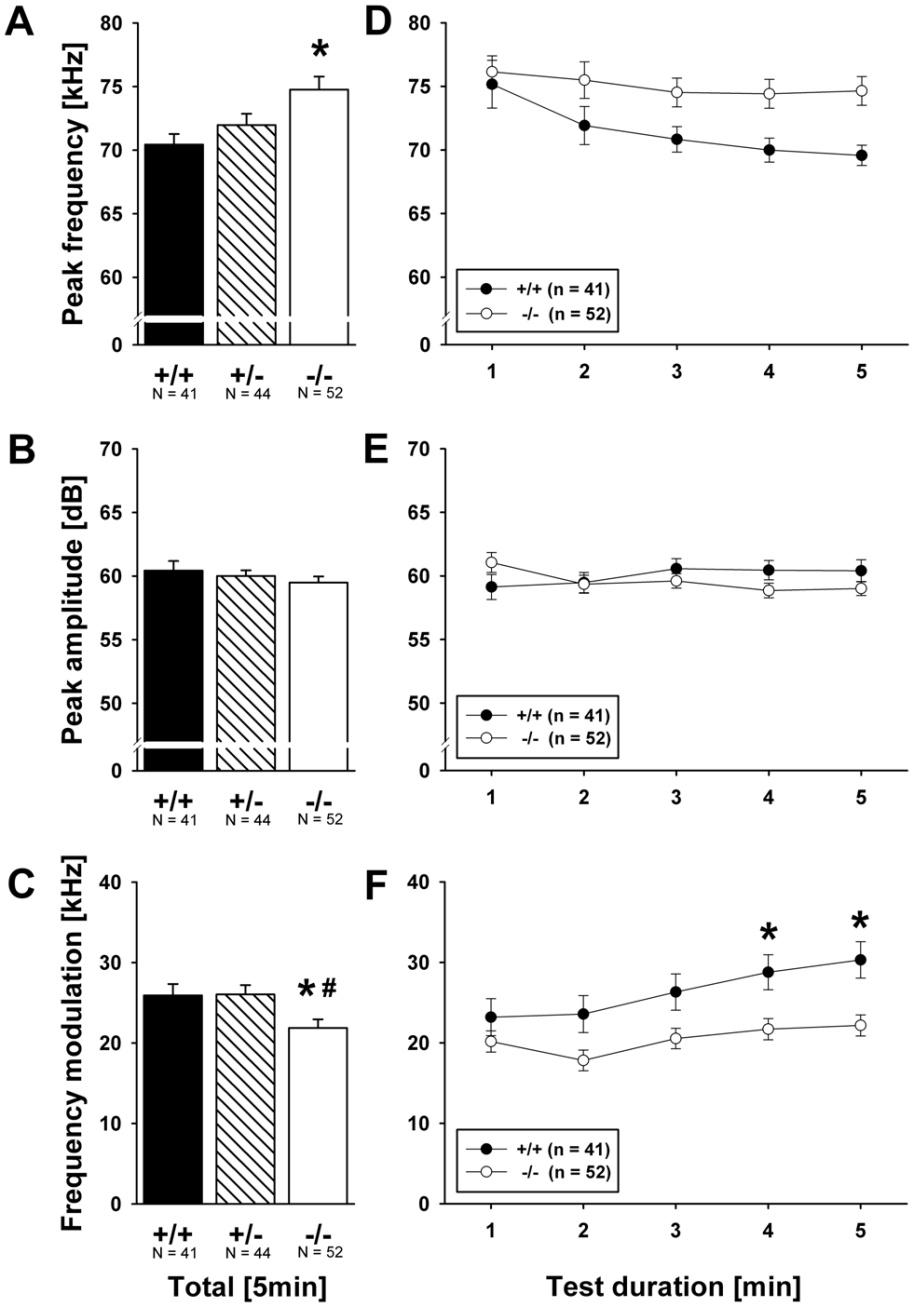
Ultrasonic vocalizations in isolated *Shank1* pups. (A) Peak frequency, (B) peak amplitude and (C) frequency modulation of calls emitted during the 5 min isolation from mother and littermates. (D) Time course for the peak frequency, (E) peak amplitude, and (F) frequency modulation of calls emitted for each 1 min time bin across the 5 min isolation session. Black bar: *Shank1^+/+^* wildtype littermate control mice; striped bar: *Shank1^+/^* ^−^ heterozygote mice; white bar: *Shank1* ^−*/*−^ null mutant mice. For the sake of clarity, *Shank1^+/^* ^−^ heterozygote mice were not included in the time course graphs, while still included in the statistical analysis. Data are presented as means ± standard errors of the mean. * p<0.050 vs. *Shank1^+/+^*; # p<0.050 vs. *Shank1^+/^*.

A genotype difference was detected in the number of USV emitted (F_2,137_ = 3.638, p = 0.029; Fig. 2A). *Shank1* ^−*/*−^ mutant pups emitted fewer USV than *Shank1^+/+^* littermate control pups (p = 0.030; all other p-values >0.050). Total calling time was also affected by genotype (F_2,137_ = 3.160, p = 0.046; Fig. 2B). *Shank1* ^−*/*−^ mutant pups spent less time calling than *Shank1^+/^* ^−^ littermates (p = 0.038; all other p-values >0.050). As there was no genotype difference in the latency to start calling (F_2,137_ = 2.281, p = 0.106) and duration of calls (F_2,137_ = 3.160, p = 0.147; Fig. 2C), this indicates a reduced call repetition rate in *Shank1* ^−*/*−^ mutant pups. Furthermore, genotype affected peak frequency of calls (F_2,137_ = 5.316, p = 0.006; Fig. 3A). *Shank1* ^−*/*−^ mutant pups emitted USV that had higher peak frequencies than USV emitted by *Shank1^+/+^* littermate control pups (p = 0.003; all other p-values >0.050). Peak amplitude of calls, i.e. loudness, was not affected (F_2,137_ = 1.509, p = 0.225; Fig. 3B). Finally, call frequency modulation differed between genotypes (F_2,137_ = 3.109, p = 0.048; Fig. 3C). *Shank1* ^−*/*−^ mutant pups emitted USV that were less frequency modulated than USV emitted by *Shank1^+/^* ^−^ (p = 0.015) and *Shank1^+/+^* littermate control pups (p = 0.022), while the latter did not differ from each other (p = 0.997).

**Figure 4:**
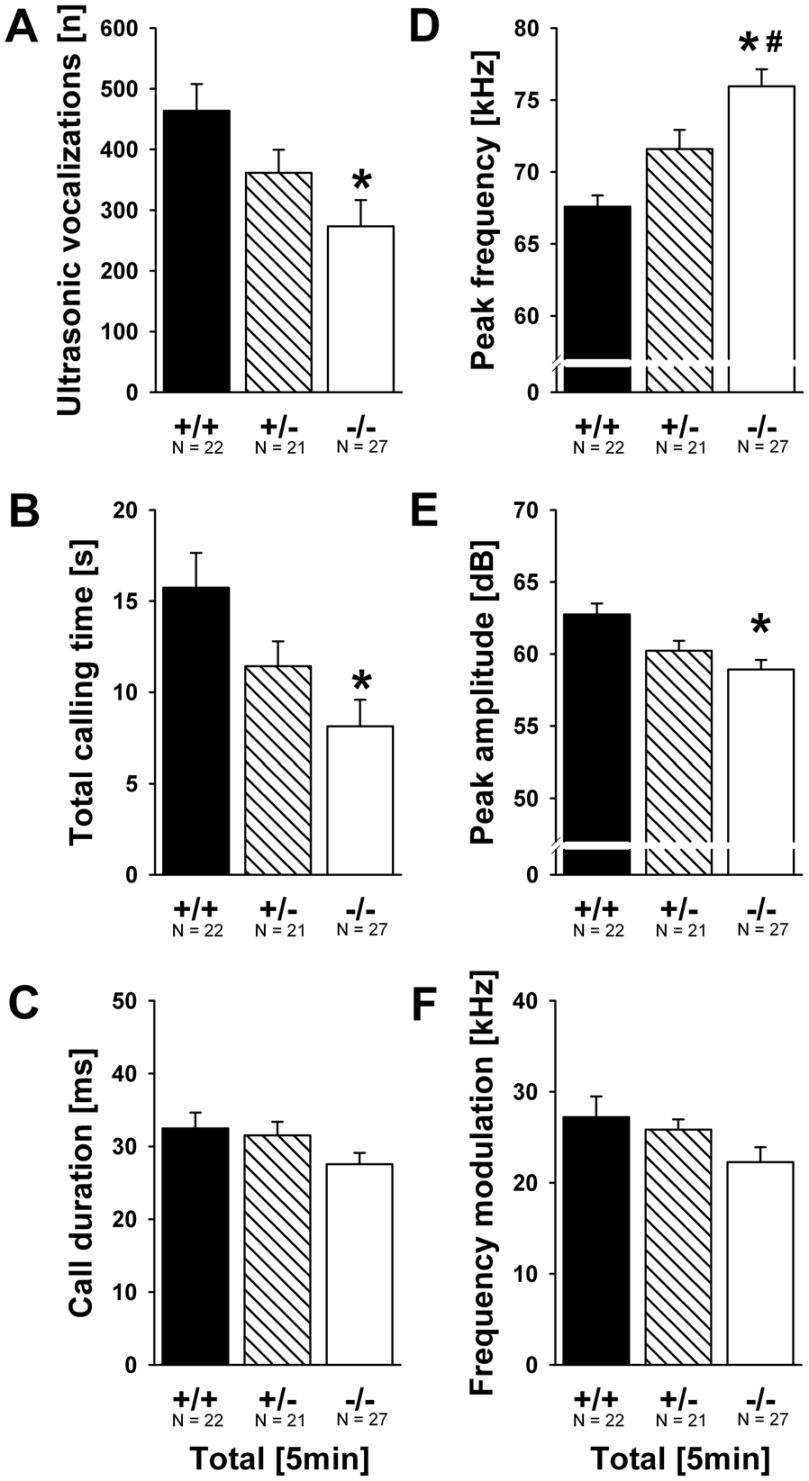
Ultrasonic vocalizations in isolated female *Shank1* pups. (A) Total number of ultrasonic vocalizations, (B) total calling time, (C) duration of calls, (D) peak frequency, (E) peak amplitude and (F) frequency modulation of calls emitted during the 5 min isolation from mother and littermates. Black bar: *Shank1^+/+^* wildtype littermate control mice; striped bar: *Shank1^+/^* ^−^ heterozygote mice; white bar: *Shank1* ^−*/*−^ null mutant mice. Data are presented as means ± standard errors of the mean. * p<0.050 vs. *Shank1^+/+^*; # p<0.050 vs. *Shank1^+/^* ^−^.

**Figure 5:**
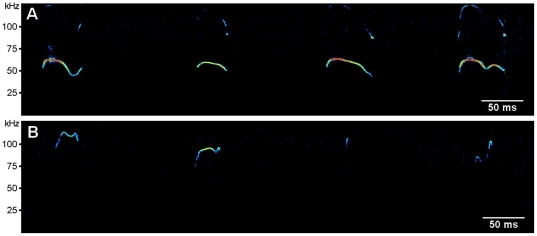
Ultrasonic vocalizations in isolated female *Shank1* pups. (A) Representative spectrograms of ultrasonic vocalizations emitted by a female *Shank1^+/+^* wildtype littermate control mouse and (B) a female *Shank1* ^−*/*−^ null mutant mouse.

**Figure 6:**
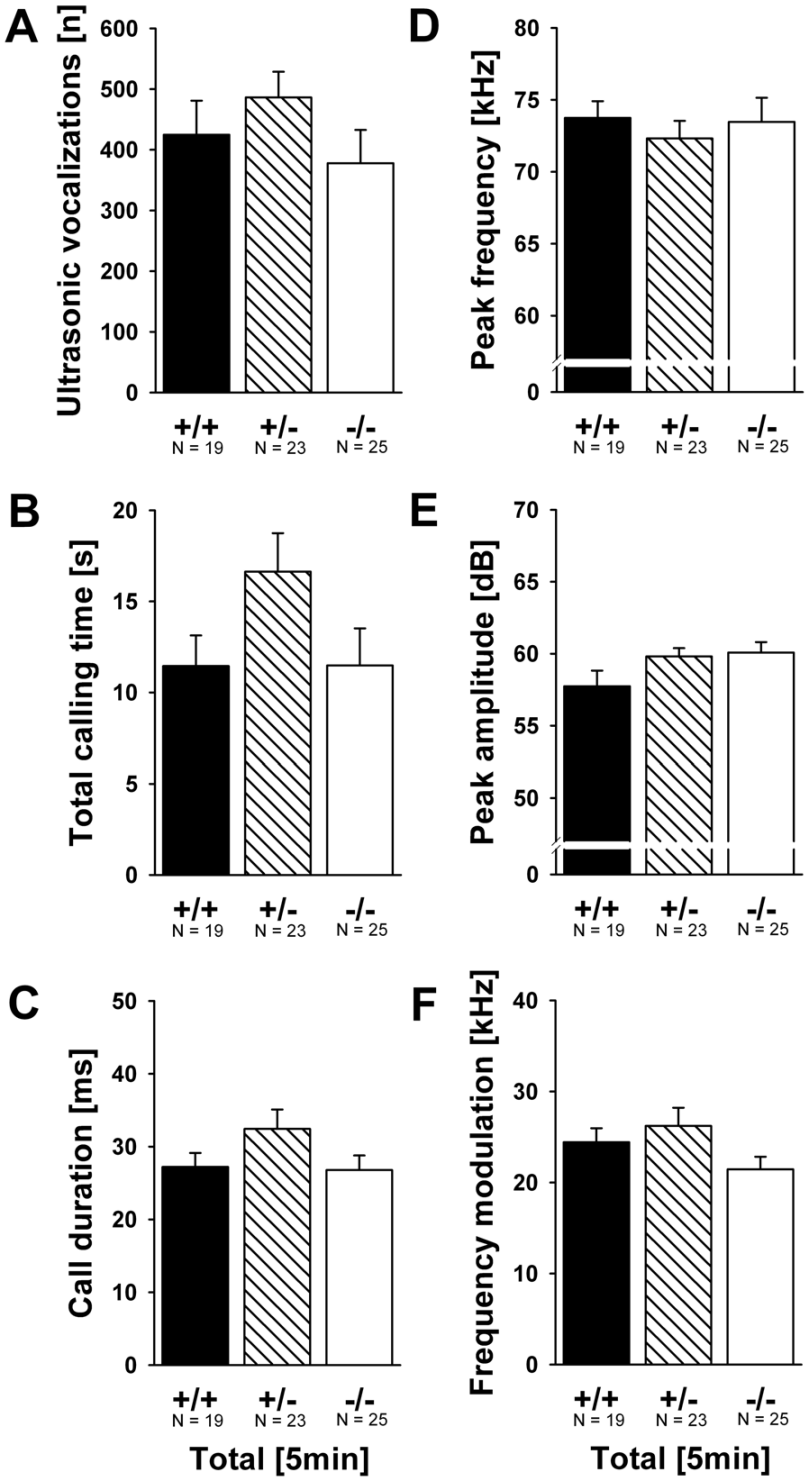
Ultrasonic vocalizations in isolated male *Shank1* pups. (A) Total number of ultrasonic vocalizations, (B) total calling time, (C) duration of calls, (D) peak frequency, (E) peak amplitude and (F) frequency modulation of calls emitted during the 5 min isolation from mother and littermates. Black bar: *Shank1^+/+^* wildtype littermate control mice; striped bar: *Shank1^+/^* ^−^ heterozygote mice; white bar: *Shank1* ^−*/*−^ null mutant mice. Data are presented as means ± standard errors of the mean. * p<0.050 vs. *Shank1^+/+^*; # p<0.050 vs. *Shank1^+/^* ^−^.

No differences were detected between male and female pups on the emission of USV (all p-values >0.050). However, there were interactions between sex and genotype for total calling time (F_2,137_ = 3.367, p = 0.038), peak frequency of calls (F_2,137_ = 4.957, p = 0.008), and peak amplitude of calls (F_2,137_ = 6.637, p = 0.002; all other p-values >0.050). For all three call parameters, this interaction is due to the fact that genotype differences were evident only in females and not in males. In females, genotype effects were detected for number of USV emitted (F_2,69_ = 5.209, p = 0.008; Fig. 4A), total calling time (F_2,69_ = 5.937, p = 0.004; Fig. 4B), peak frequency (F_2,69_ = 14.276, p<0.001; Fig. 4D) and peak amplitude (F_2,69_ = 7.763, p = 0.001; Fig. 4E), while latency to start calling, duration of calls and call frequency modulation were not affected (all p-values >0.050; Fig. 4C & 4F; representative spectrograms: Fig. 5). Female *Shank1* ^−*/*−^ mutant pups emitted fewer USV than female *Shank1^+/+^* littermate control pups (p = 0.005; all other p-values >0.050), resulting in a lower total calling time in the former (p = 0.003; all other p-values >0.050). Furthermore, female *Shank1* ^−*/*−^ mutant pups emitted USV that had higher peak frequencies than USV emitted by female *Shank1^+/^* ^−^ (p = 0.021) and *Shank1^+/+^* littermate control pups (p<0.001), while the latter did not differ (p>0.050). Finally, female *Shank1* ^−*/*−^ mutant pups emitted USV that had lower peak amplitudes than USV emitted by female *Shank1^+/+^* littermate control pups (p = 0.001; all other p-values >0.050). In males, no genotype effects were detected (all p-values >0.050; Fig. 6).

An effect of parity, i.e. first versus second litter, was detected in the latency to start calling (F_1,137_ = 4.569, p = 0.035), peak amplitude of calls (F_1,137_ = 13.889, p<0.001), and call frequency modulation (F_1,137_ = 40.164, p<0.001; all other p-values >0.050). First litter mice started to emit USV earlier and their calls were lower in amplitude, but more frequency modulated as compared to second litter mice. No interactions between parity and genotype or sex were detected (all p-values >0.050).

Across the one minute time bins in the five minute isolation test, increasing numbers of calls were detected (F_4,500_ = 10.586, p<0.001, min 1 vs. min 5, p = 0.003; Fig. 2D). This increase in call number tended to be genotype-dependent (F_4,500_ = 1.867, p = 0.063). While there was an increase over time during testing in number of calls emitted in *Shank1^+/+^* littermate control pups (min 1 vs. min 5: p = 0.013), no such increase was detected in *Shank1* ^−*/*−^ mutant pups (min 1 vs. min 5: p>0.999), resulting in genotype differences in min 4 (p = 0.023) and min 5 (p = 0.003; p-values for all other min >0.050). Similarly, the time spent vocalizing increased across testing (F_4,500_ = 32.352, p<0.001, min 1 vs. min 5, p<0.001; Fig. 2E) in a genotype-dependent manner (F_4,500_ = 2.414, p = 0.015). While there was an increase over time during testing in the time spent calling in *Shank1^+/+^* littermate control pups (min 1 vs. min 5: p<0.001), no such increase was detected in *Shank1* ^−*/*−^ mutant pups (min 1 vs. min 5: p = 0.407), resulting in a genotype difference in min 5 (p = 0.040; p-values for all other min >0.050). Call duration increased across testing (F_4,500_ = 100.083, p<0.001, min 1 vs. min 5, p<0.001; Fig. 2F). Again, this increase was affected by genotype (F_4,500_ = 2.484, p = 0.012). While call duration increased in both genotypes, the increase was more pronounced in *Shank1^+/+^* (min 1 vs. min 5: p<0.001) than in *Shank1* ^−*/*−^ pups (min 1 vs. min 5: p<0.001), but no genotype differences were detected (p-values for all min >0.050). Peak frequency of calls decreased across the one minute time bins in the five minute isolation test (F_4,500_ = 8.898, p<0.001, min 1 vs. min 5, p = 0.001; Fig. 3D), but this decrease was not dependent on genotype (F_4,500_ = 1.4087, p = 0.191). Peak amplitude of calls did not change across testing and no effect of genotype was found thereon (all p-values >0.050; Fig. 3E). Finally, call frequency modulation increased over time (F_4,500_ = 18.471, p<0.001, min 1 vs. min 5, p<0.001; Fig. 3F), which was genotype-dependent (F_4,500_ = 2.103, p = 0.034). While there was an increase across testing in call frequency modulation in *Shank1^+/+^* littermate control pups (min 1 vs. min 5: p = 0.003), no such increase was detected in *Shank1* ^−*/*−^ mutant pups (min 1 vs. min 5: p = 0.284), resulting in a genotype difference in min 4 (p = 0.042) and min 5 (p = 0.043; p-values for all other min >0.050). Notably, changes in the USV emission over time during testing were not dependent on sex or parity (all p-values >0.050).

### Body weight and body temperature in isolated pups

**Figure 7:**
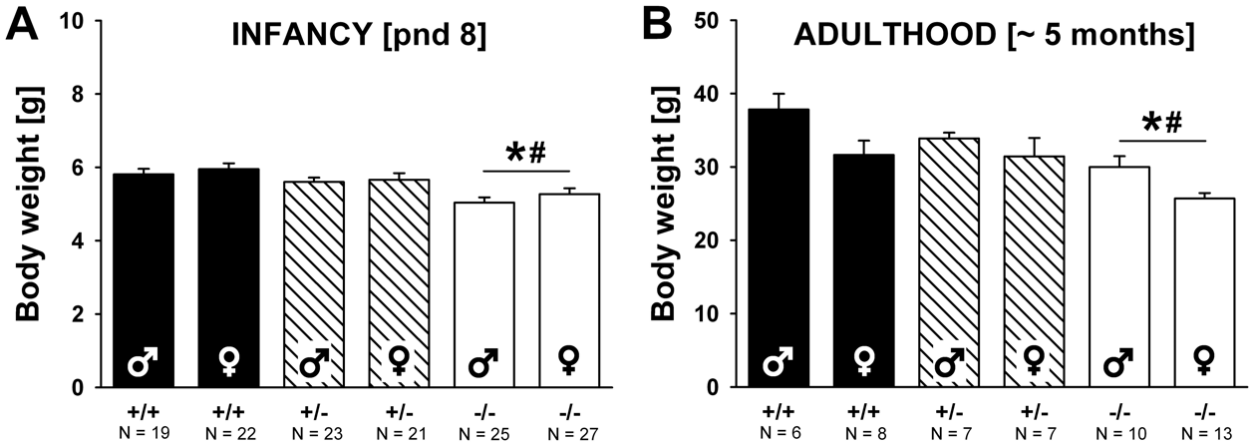
Body weight in *Shank1* mice. (A) Body weight in pups tested for isolation-induced ultrasonic vocalizations on postnatal day (pnd) 8. (B) Body weight in adult mice approximately 5 months of age. Black bar: *Shank1^+/+^* wildtype littermate control mice; striped bar: *Shank1^+/^* ^−^ heterozygote mice; white bar: *Shank1* ^−*/*−^ null mutant mice. Data are presented as means ± standard errors of the mean. * p<0.050 vs. *Shank1^+/+^*; # p<0.050 vs. *Shank1^+/^* ^−^.

In the mouse pups tested for USV in isolation, body weight differed between genotypes (F_2,137_ = 10.389, p<0.001; Fig. 7A). *Shank1* ^−*/*−^ mutant pups were lighter than *Shank1^+/^* ^−^ (p = 0.006) and *Shank1^+/+^* littermate control pups (p<0.001), while the latter did not differ from each other (p = 0.251). Body temperature was not affected by genotype (F_2,137_ = 2.910, p = 0.058). Body weight and body temperature were not correlated with the number of USV emitted (r = −0.124, p = 0.148 and r = −0.130, p = 0.129, respectively) or other USV characteristics, with the exception of a very low negative correlation between body weight and total calling time (r = −0.171, p = 046; all other p-values >0.050). Sex had no effect on body weight or body temperature (all p-values >0.050). Parity affected body weight (F_2,137_ = 4.094, p = 0.045), but not body temperature (F_2,137_ = 0.001, p = 0.984). First litter mice were heavier than second litter mice.

Lower body weights in *Shank1* ^−*/*−^ mice persisted at approximately 5 months of age (F_1,38_ = 22.029, p<0.001; Fig. 7B). *Shank1* ^−*/*−^ mutant pups were lighter than *Shank1^+/^* ^−^ (p = 0.005) and *Shank1^+/+^* littermate control pups (p<0.001), while the latter did not differ from each other (p = 0.605). In adulthood, a clear effect of sex was found, with males heavier than females (F_1,38_ = 12.843, p = 0.001), as expected.

### Open field activity in the absence and presence of female urine in adult males

**Figure 8:**
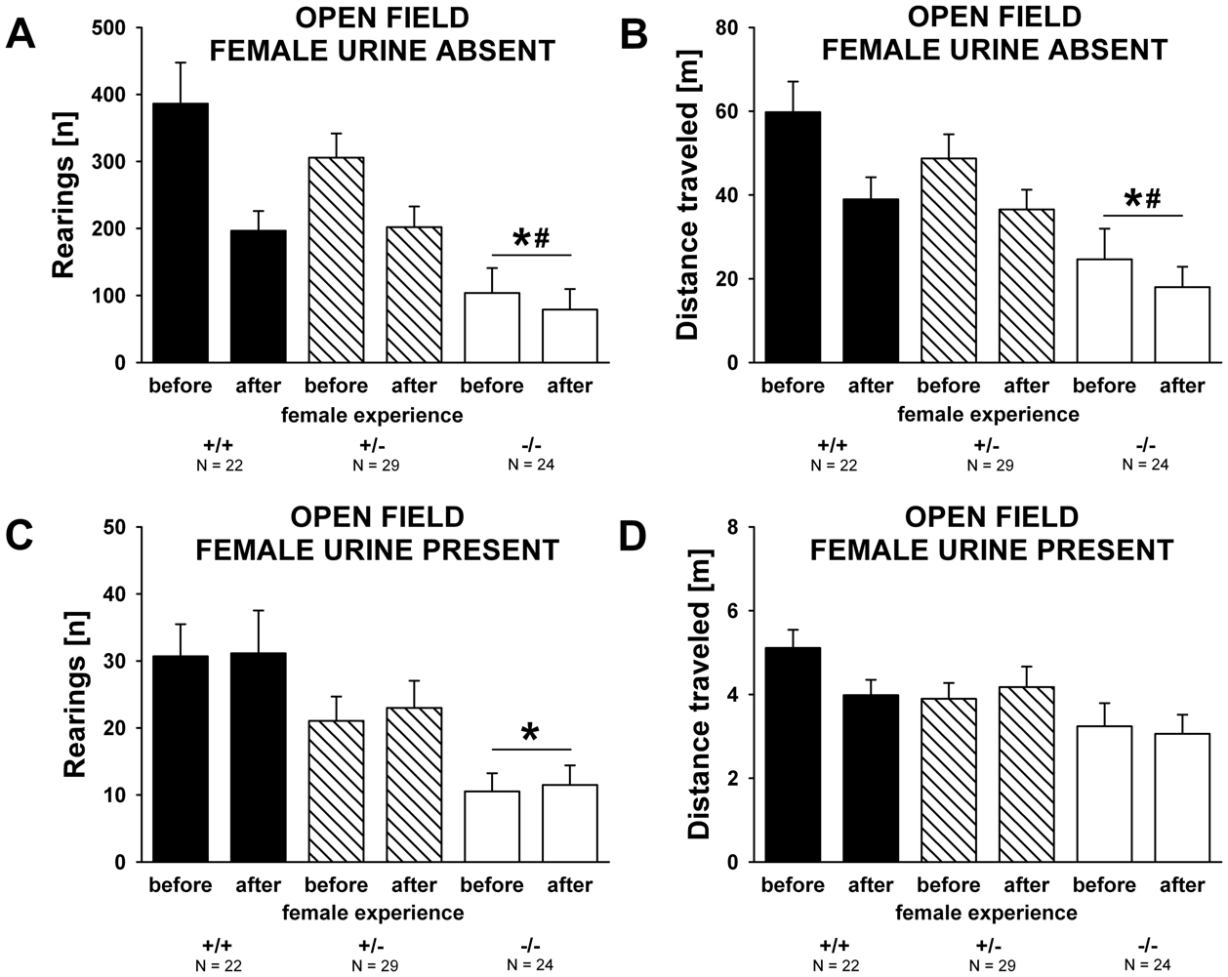
Open field activity in the absence and presence of female urine in adult male *Shank1* mice. (A) Total number of rearings and (B) distance traveled during the 60 min habituation session to the clean open field without female urine displayed by male subjects before they had an experience of social interactions with a female, and 7 days after they had a 5 minute experience of social interactions with a female. (C) Total number of rearings and (D) distance traveled during the 5 min test session in the same open field containing urine from a female C57BL/6J mouse displayed by male subjects before they had an experience of social interactions with a female and after female experience. Black bar: *Shank1^+/+^* wildtype littermate control mice; striped bar: *Shank1^+/^* ^−^ heterozygote mice; white bar: *Shank1* ^−*/*−^ null mutant mice. Data are presented as means ± standard errors of the mean. * p<0.050 vs. *Shank1^+/+^*; # p<0.050 vs. *Shank1^+/^* ^−^.

When comparing the locomotor activity shown by adult males exposed to the clean open field without female urine before and after female experience, a reduction in number of rearings and distance traveled was found, probably reflecting habituation (F_1,72_ = 32.817, p<0.001 and F_1,72_ = 27.297, p<0.001, respectively; Fig. 8A & 8B). Interestingly, no reduction in number of rearings and distance traveled was observed when comparing the locomotor activity shown by males after adding the female urine spot before and after female experience, indicating an inhibition of the habituation effect in the presence of the female urine spot (all p-values >0.050; Fig. 8C & 8D).

There was a genotype effect on rearing behavior when males were initially exposed to the clean open field without female urine (F_2,72_ = 8.973, p = 0.001; Fig. 8A). *Shank1* ^−*/*−^ mutant mice displayed a lower number of rearings, when tested in the clean open field before and after female experience, as compared to *Shank1^+/^* ^−^ (p = 0.003) and *Shank1^+/+^* littermate control mice (p = 0.001). *Shank1^+/^* ^−^ and *Shank1^+/+^* mice did not differ from each other (p = 0.724). Distance traveled differed between genotypes (F_2,72_ = 6.716, p = 0.002; Fig. 8B). The distance traveled by *Shank1* ^−*/*−^ mutant mice was lower before and after female experience than the distance traveled by *Shank1^+/^* ^−^ (p = 0.017) and *Shank1^+/+^* littermate control mice (p = 0.003), while the latter did not differ from each other (p = 0.664). These genotype differences in locomotor activity were still evident after adding the female urine spot to the open field. Again, there was a genotype effect on rearing behavior (F_2,72_ = 6.936, p = 0.002; Fig. 8C). *Shank1* ^−*/*−^ mutant mice displayed a lower number of rearings before and after female experience than *Shank1^+/+^* littermate control mice (p = 0.001; all other p-values >0.050). There was a trend for a genotype difference in the distance traveled (F_2,72_ = 2.939, p = 0.059; Fig. 8D). In addition to these main effects of genotype, evidence for interactions between genotype and female experience was obtained. The reduction in number of rearings and distance traveled seen in males exposed to the clean open field without female urine when comparing before and after female experience was less pronounced in *Shank1* ^−*/*−^ than in *Shank1^+/^* ^−^ and *Shank1^+/+^* mice (F_2,72_ = 6.138, p = 0.003 and F_2,76_ = 2.461, p = 0.093; respectively). No such interactions were obtained in males exposed to the open field after adding female urine (all p-values >0.050).

### Scent marking behavior in the absence and presence of female urine in adult males

**Figure 9:**
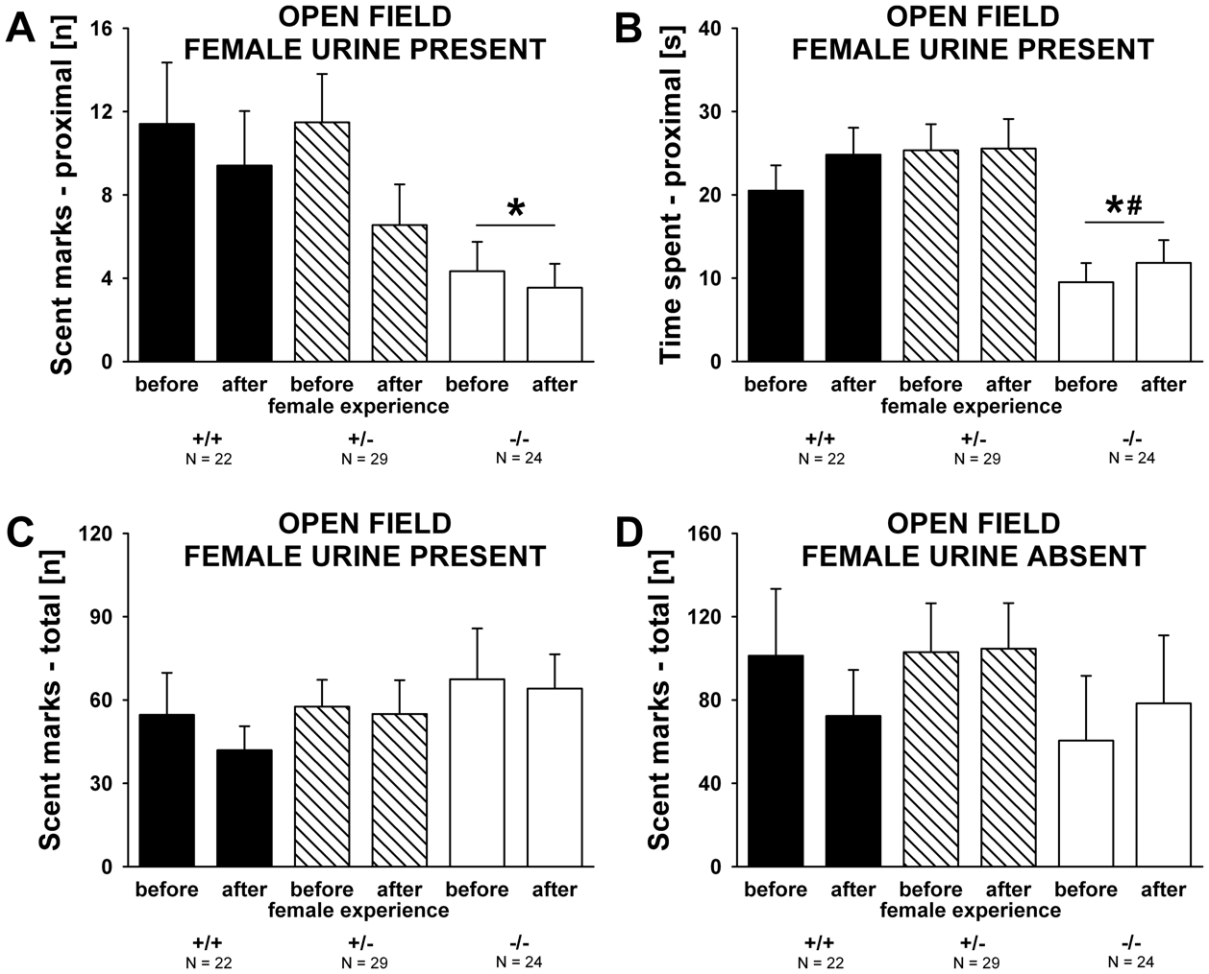
Scent marking behavior in the absence and presence of female urine in adult male *Shank1* mice. (A) Number of scent marks deposited near (within 10 cm^2^ around) the female urine spot deposited by male subjects before they had an experience of social interactions with a female, and 7 days after they had a 5 minute experience of social interactions with a female. (B) Time spent in proximity to the female urine spot (10 cm^2^) by male subjects before they had an experience of social interactions with a female and after female experience. (C) Total number of scent marks deposited throughout the entire open field during the 5 min test session in the open field containing urine from a female C57BL/6J mouse deposited by male subjects before they had an experience of social interactions with a female and after female experience. (D) Total number of scent marks deposited throughout the entire open field during the 60 min habituation session in the clean open field without female urine deposited by male subjects before they had an experience of social interactions with a female and after female experience. Black bar: *Shank1^+/+^* wildtype littermate control mice; striped bar: *Shank1^+/^* ^−^ heterozygote mice; white bar: *Shank1* ^−*/*−^ null mutant mice. Data are presented as means ± standard errors of the mean. * p<0.050 vs. *Shank1^+/+^*; # p<0.050 vs. *Shank1^+/^* ^−^.

Genotypes differed on scent marking behavior in the proximity to the female urine spot (F_2,72_ = 3.399, p = 0.039; Fig. 9A). *Shank1* ^−*/*−^ mutant mice deposited fewer urine traces in proximity to the female urine spot, in scent marking tests conducted both before and after female experience, as compared to *Shank1^+/+^* littermate control mice (p = 0.045; all other p-values >0.050). Time spent in proximity to the female urine spot showed similar genotype effects (F_2,72_ = 8.937, p<0.001; Fig. 9B). *Shank1* ^−*/*−^ mice spent less time within the area of 10 cm^2^ around the female urine spot than *Shank1^+/^* ^−^ (p<0.001) and *Shank1^+/+^* littermate control mice (p = 0.008), while the latter did not differ from each other (p = 0.738). There was no genotype difference in the number of urine traces deposited in the entire open field in the presence of female urine, nor in the absence of female urine when males were initially exposed to the clean open field (F_2,72_ = 0.597, p = 0.553 and F_2,72_ = 0.514, p = 0.600, respectively; Fig. 9C & 9D). Prior experience with a female did not affect scent marking behavior and no evidence for an interaction between genotype x female experience was obtained (all p-values >0.050).

### Ultrasonic vocalizations in the presence of female urine in adult males

**Figure 10:**
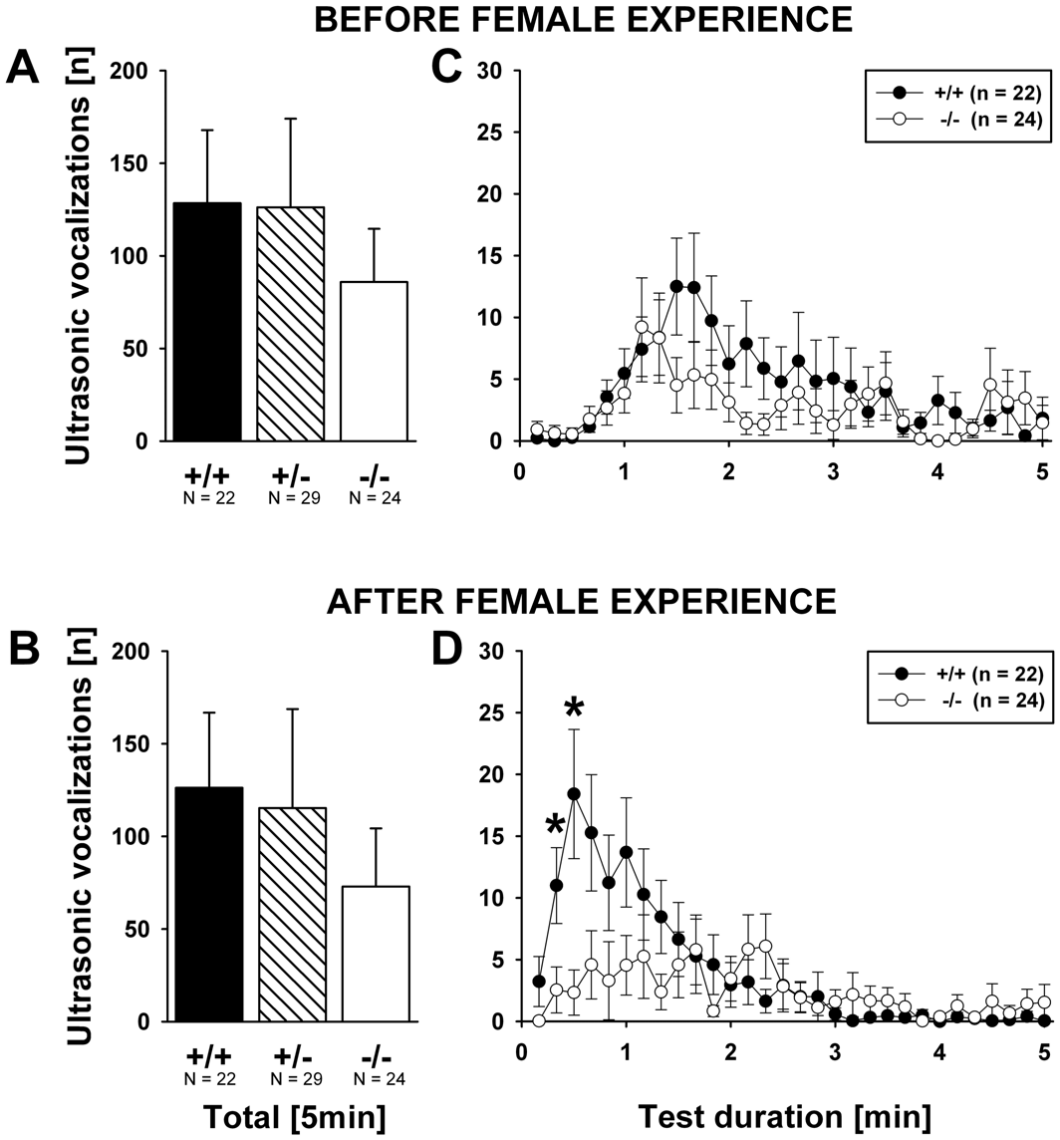
Ultrasonic vocalizations in the presence of female urine in adult male *Shank1* mice. (A) Total number of ultrasonic vocalizations emitted during the 5 min test session in the open field containing urine from a female C57BL/6J mouse by male subjects before they had an experience of social interactions with a female. (B) Total number of ultrasonic vocalizations emitted during the 5 min test session in the open field containing urine from a female C57BL/6J mouse by male subjects 7 days after they had a 5 minute experience of social interactions with a female. (C) Time course for the number of ultrasonic vocalizations emitted for each 10 s time bin across the 5 min test session with exposure to female urine before female experience. (D) Time course for the number of ultrasonic vocalizations emitted for each 10 s time bin across the 5 min test session with exposure to female urine after female experience. Black bar: *Shank1^+/+^* wildtype littermate control mice; striped bar: *Shank1^+/^* ^−^ heterozygote mice; white bar: *Shank1* ^−*/*−^ null mutant mice. For the sake of clarity, *Shank1^+/^* ^−^ heterozygote mice were not included in the time course graphs, while still included in the statistical analysis. Data are presented as means ± standard errors of the mean. * p<0.050 vs. *Shank1^+/+^*; # p<0.050 vs. *Shank1^+/^* ^−^.

The number of USV emitted by males during the entire 5 min exposure to the female urine spot did not differ when subjects were tested before versus after female experience (F_1,72_ = 0.317, p = 0.575) and was not dependent on genotype (F_2,72_ = 0.387, p = 0.680; Fig. 10A & 10B). No evidence for an interaction between female experience and genotype was found (F_2,72_ = 0.042, p = 0.959).

However, when the time course of USV emission was taken into account, an inverted U-shaped calling pattern became apparent (F_29,2088_ = 10.151, p<0.001) that was dependent on female experience (F_29,2088_ = 6.589, p<0.001) as a faster onset of the USV response was seen after female experience. Most importantly, the change in the time course of USV emission that was seen after female experience was dependent on genotype (F_58,2088_ = 1.730, p = 0.001; all other p-values >0.050). Specifically, while a slightly inverted U-shaped calling pattern was seen in both genotypes before female experience, *Shank1^+/+^* mice displayed a clearly inverted U-shaped calling pattern characterized by a fast onset response after female experience. In contrast, however, a lack of such an inverted calling pattern was seen in *Shank1* ^−*/*−^ mice. Indeed, when comparing the time course of female urine-elicited USV in *Shank1^+/+^* mice before and after female experience, a more pronounced inverted U-shaped call pattern with a clear shift towards the beginning of testing was evident after female experience (F_29,609_ = 4.516, p<0.001). The calling pattern of *Shank1* ^−*/*−^ mice did not differ before and after female experience (F_29,667_ = 1.194, p = 0.223). Accordingly, there was no difference in the calling pattern between *Shank1* ^−*/*−^ and *Shank1^+/+^* mice before female experience (F_29,1276_ = 1.090, p = 0.340; Fig. 10C), but after female experience (F_29,1276_ = 3.747, p<0.001; Fig. 10D), resulting in a genotype difference in min 1 (second time bin: p = 0.021; third time bin: p = 0.004; all p-values for other time bins >0.050). This indicates that *Shank1^+/+^* mice changed their calling pattern dependent on female experience, while *Shank1* ^−*/*−^ mice did not change their calling pattern as a consequence of prior experience with a female.

## Discussion

USV emission [28]–[31] and the deposition of scent marks [32]–[34] appear to be the two major modes of mouse social communication. Assays for USV and scent marking behavior may be useful for evaluating communication abilities in mouse models of autism [48]–[62], [71], [76], [80]–[82], [102]. We addressed the possibility that USV emission and scent marking behavior can be simultaneously assayed to evaluate communication deficits in mice with mutations in candidate genes for autism. Although no cases of mutations in *SHANK1* have yet been identified in individuals with autism, *SHANK1* is a member of the *SHANK* gene family, in which mutations in *SHANK2* and *SHANK3* have been detected in several autistic individuals [6]–[11].

Our findings revealed deficits in several elements of social communication and early developmental milestones in mice with a null mutation in *Shank1*. As pups, *Shank1* ^−*/*−^ mutant mice emitted fewer USV than *Shank1^+/+^* littermate control mice when isolated from mother and littermates. Call characteristics and their changes over time during testing differed between *Shank1* ^−*/*−^ and *Shank1^+/+^* mice. As adults, both *Shank1^+/+^* and *Shank1* ^−*/*−^ male mice emitted a similar amount of USV when exposed to female urine. Importantly, however, *Shank1^+/+^* adult males changed their calling pattern dependent on their previous exposure to a female, but *Shank1* ^−*/*−^ adult males were unaffected by prior female experience. In addition, scent marking behavior in the presence of female urine, but not in the absence of female urine, was affected by genotype. Specifically, *Shank1* ^−*/*−^ mutant mice deposited fewer urine traces in proximity to the female urine spot than *Shank1^+/+^* littermate control mice. Besides these differences in USV emission and scent marking behavior, reduced levels of locomotor behavior were observed in *Shank1* ^−*/*−^ mutant mice.

Motor impairments have previously been reported in this line of *Shank1* mutant mice. When tested on an accelerating rotarod and in a wire hang task, the latency to fall was lower in *Shank1* ^−*/*−^ than in *Shank1^+/+^* mice [22], [23]. Consistent with deficits in motor performance, we found that the surface righting reflex, which is predominantly dependent on body righting mechanisms, strength, and coordination, was delayed in *Shank1* ^−*/*−^ as compared to *Shank1^+/+^* mice. Body weight gain and the appearance of some physical landmarks, pinnae detachment and incisor eruption, were delayed in *Shank1* ^−*/*−^ mice. Our findings further replicated a previously reported open field activity deficit [22], [23]. Whether *Shank1* ^−*/*−^ mutant mice were exposed to a novel or a familiar open field, and whether female urine was present or not, they displayed fewer rearings and their distance traveled was lower than in *Shank1^+/+^* littermate controls.

When isolated from mother and littermates on postnatal day 8, *Shank1* ^−*/*−^ mutant pups emitted fewer USV than *Shank1^+/+^* littermate control pups, a finding which could indicate an early communication deficit in *Shank1* ^−*/*−^ mice. As there was no genotype difference in the latency to start calling, these data represent a lower call repetition rate in *Shank1* ^−*/*−^ than *Shank1^+/+^* pups. Calls emitted by *Shank1* ^−*/*−^ were higher in peak frequency and less frequency modulated than the ones emitted by *Shank1^+/+^* pups. Changes in USV emission over testing duration were highly dependent on genotype. While there was an increase in call number, total calling time, duration, and frequency modulation of calls in *Shank1^+/+^* mice, this increase was weaker or absent in *Shank1* ^−*/*−^ mice. Therefore, all call parameters, with the exception of peak amplitude, i.e. loudness, were either directly affected by genotype, or their temporal pattern was affected by genotype. This is particularly remarkable as the two other factors studied, sex and parity, i.e. litter order, had only minor effects on USV production.

Genotype affected isolation-induced USV primarily in females. This is surprising given the typical 4:1 male:female ratio in autism. It is therefore of particular interest that such a male bias was also not found in human autism studies on mutations in *SHANK2* and *SHANK3*. As in the present study, a female bias was reported instead [6], [8], [10]. In order to test whether communication deficits found in female *Shank1* ^−*/*−^ pups persist into adulthood, we currently assess USV emitted during social interactions of adult females as such USV reflect the level of social interest and serve an important communicative function [103], [104].

Since a genotype difference was detected for body weight, it is possible that the lower USV level in *Shank1* ^−*/*−^ mice is due to their lower body weight. In light of the small genotype difference in body weight of only 0.75 grams, however, body weight appears unlikely to be the cause of the reduced USV level in *Shank1* ^−*/*−^ pups. Lack of correlation between body weight and USV emission further argues against an interpretation that genotype differences in call emission were due to differences in body weight.

Another potentially confounding factor is a genotype difference in anxiety-related behavior. *Shank1* ^−*/*−^ null mutant mice displayed higher levels of anxiety-related behavior on some components of the light/dark exploration test as compared to *Shank1^+/+^* mice [22], [23], although elevated plus maze scores did not differ across genotypes [23]. Isolation-induced USV can be enhanced by anxiogenic substances, while anxiolytic substances reduce calling levels, supporting the notion that isolation-induced USV reflect a negative affective state akin to anxiety or high stress reactivity [105]–[109]. However, it appears unlikely that the genotype difference in pup USV is due to a difference in anxiety levels, since one would have expected more, but not less USV in *Shank1* ^−*/*−^ mice that display higher levels of anxiety-related behavior.

There is compelling evidence that isolation-induced USV serve a communicative function. Pup calls elicit maternal search and retrieval behavior, as shown in playback experiments [36–31]. A reduced level of calling or an unusual calling pattern has been reported in several mouse models of autism [48]–[62], which could be indicative of a communication impairment. Importantly, it was shown that less maternal caregiving was directed to mouse pups that vocalized only rarely [110]. Hung et al. [22] reported a high rate of death in pups bred from homozygous matings of *Shank1* ^−*/*−^ males and females. It is tempting to speculate that some of the mouse pups bred by using a homozygous breeding regimen were not nurtured, and hence died before weaning, because the reduced level of call production in *Shank1* ^−*/*−^ mouse pups was insufficient to elicit maternal care.

It appears possible that other call parameters such as call duration, peak frequency, peak amplitude, and frequency modulation, affect the communicative value of USV. Playback studies have shown that lactating mice can distinguish between different call types, and that they prefer certain call types over others if given the choice [37], [38], [40]. Smith [40] showed that mothers prefer a call with an 80 ms duration over a call with a 15 ms duration. Ehret [37] found that mothers respond to calls with durations higher than 30 ms, but not to shorter ones. With respect to peak frequency, mothers showed a stronger response towards a 65–45 kHz signal than to a 75–55 kHz signal [40]. This is probably because the mouse auditory thresholds increase rapidly above 60 kHz [111]. Call amplitude seems also to be important to attract the mother. By means of a pup discrimination task where two vocalizing pups were presented, it was shown that mothers spent more time near pups emitting loud calls [41]. Finally, Brudzynski et al. [112] postulated that the level of frequency modulation could be important for the efficacy of maternal search and retrieval behavior. It may be easier for the mother to detect and localize a highly frequency modulated call than a steady sound at a constant frequency. Calls emitted by *Shank1* ^−*/*−^ mouse pups were shorter, higher in peak frequency, but less frequency modulated than the ones emitted by *Shank1^+/+^* pups. This means that all altered parameters of calls emitted by *Shank1* ^−*/*−^ mouse pups may decrease their signal value and hence elicit less maternal caregiving responses.

In support of a lifelong communication impairment, USV emission in adulthood was also affected by a lack of *Shank1*. In particular, the time course of the emission was dependent on genotype. Whereas *Shank1^+/+^* emitted high numbers of calls to a spot of urine from a female mouse during the first two minutes of the test session, *Shank1* ^−*/*−^ mutant mice emitted remarkably few calls during the first two minutes of the test session. Intriguingly, *Shank1^+/+^* mice changed their calling pattern dependent on a prior experience interacting with a female, but the calling pattern of *Shank1* ^−*/*−^ mice was unaffected by female experience. The experience-dependent change in calling pattern by *Shank1^+/+^* mice is a typical phenomenon in mice, which has been repeatedly replicated [64], [65], [67], [69]–[73]. Adult male mice vocalize to female urine to a greater extent after they have interacted with a live, behaving adult female mouse, indicating that a cognitive association has been formed between the social olfactory cue and other physical properties of the female. One interpretation is therefore that the lack of experience-induced changes in USV production represents an inability to modulate social behaviors in response to experiences with social cues. Shank1 proteins of the PSD promote morphological and functional maturation of dendritic spines and synapse formation [20], [113], which in turn are believed to underlie learning and memory [114], [115]. Hung et al. [22] showed that *Shank1* ^−*/*−^ mice displayed impairment of contextual fear conditioning, but normal cued fear conditioning, indicating a deficit in hippocampus-dependent memory processes. Another interpretation of the lack of experience-induced changes in USV production could therefore be related to the cognitive phenotypes reported for *Shank1* ^−*/*−^ null mutants, conceivably in the domain of social learning and memory. Finally, the weaker USV response of *Shank1* ^−*/*−^ mice in response to female urine could also be a consequence of potential social behavior abnormalities during female exposure.

Olfaction is the predominant modality for communication in many rodents including mice [32]–[34]. We employed an established scent marking task [71], [76], [83]–[87] to evaluate responses to olfactory social cues in *Shank* mice. In this task, scent marking in proximity to the female urine spot appears to be the most sensitive measure for communication deficits [71], [76]. *Shank1* ^−*/*−^ mutant mice deposited fewer urine traces in close proximity to the female urine spot as compared to *Shank1^+/+^* littermate control mice. Importantly, scent marking differences between genotypes were specifically detected in the presence of female urine but not in its absence, as genotypes did not differ on number of scent marks deposited in a clean open field. Therefore, level of urination in general was not affected by genotype. Instead, it appears that the deposition of urinary scent marks within an area of 10 cm^2^ of social odors around an aliquot of fresh urine from a female represents a species-typical response to social communicative signals, which differs in magnitude between *Shank1* ^−*/*−^ and *Shank1^+/+^* mice. No evidence for reduced levels of social interactions in *Shank1* ^−*/*−^ mutant mice was obtained on tests of juvenile reciprocal social interaction and adult social approach [23]. The present results appear to support an interpretation of a specific deficit in communicative behavior in *Shank1* ^−*/*−^ mice as deficits in USV production and scent marking behavior observed are therefore unlikely to be due to a lack of social motivation, though level of social motivation in *Shank1* ^−*/*−^ mutant mice needs to be tested formally. The social conditioned place preference test has provided a valid measure of social motivation and has been employed for studies of mice [116], [117], hamsters [118] and rats [119], [120].

While *SHANK1* has not been associated with autism, mutations in *SHANK2* and *SHANK3*, other members of the *SHANK* gene family, appear in several individuals with autism [4]–[11]. In the mouse brain, Shank protein interaction partners such as Homer or GKAP are equally recognized by all three Shank isoforms due to their structural similarity [17], [21]. Furthermore, the expression patterns of the three *Shank* isoforms in the brain are overlapping, though differences in some structures such as striatum and thalamus were described [121]–[123]. As all members of the *SHANK* gene family appear to fulfill similar physiological roles and display considerable neuroanatomical co-expression, it is intriguing that *Shank3* ^−*/*−^ null mutant mice display social deficits and repetitive self-grooming behavior [123], while no such phenotypic changes relevant to the first and third diagnostic symptom of autism were found in *Shank1* ^−*/*−^ null mutant mice [23]. Despite our findings of reduced levels of USV and scent marking behavior in social contexts that are consistent with a *Shank1* ^−*/*−^ phenotype relevant to the second diagnostic symptom of autism, communication deficits, the *Shank1* ^−*/*−^ null mutant mice do therefore not qualify for a genetic mouse model of autism, covering all three diagnostic symptoms.
